# Supplementary material for: Glucose addition promotes C fixation and bacteria diversity in C-poor soils, improves root morphology, and enhances key N metabolism in apple roots
Source: PLoS One. 2022 Jan 19;17(1):e0262691. doi: 10.1371/journal.pone.0262691 (PMC8773054; doi:10.1371/journal.pone.0262691)
Supplement: S2 Fig — Content of total nitrogen (TN) of soil (A) and ratio of total soil organic carbon to TN (C/N, B) in the sterilized and non-sterilized soils with glucose addition at day 45. CK, non-sterilized soil without glucose addition; Glu-1, non-sterilized soil with low level of glucose addition; Glu-2, non-sterilized soil with high level of glucose addition; SS, sterilized soil without glucose addition; SS+Glu-1, sterilized soil with low level of glucose addition; SS+Glu-2, sterilized soil with high level of glucose addition. Different lowercase letters indicate significant differences (P < 0.05) among treatments. (DOCX) [file pone.0262691.s002.docx]

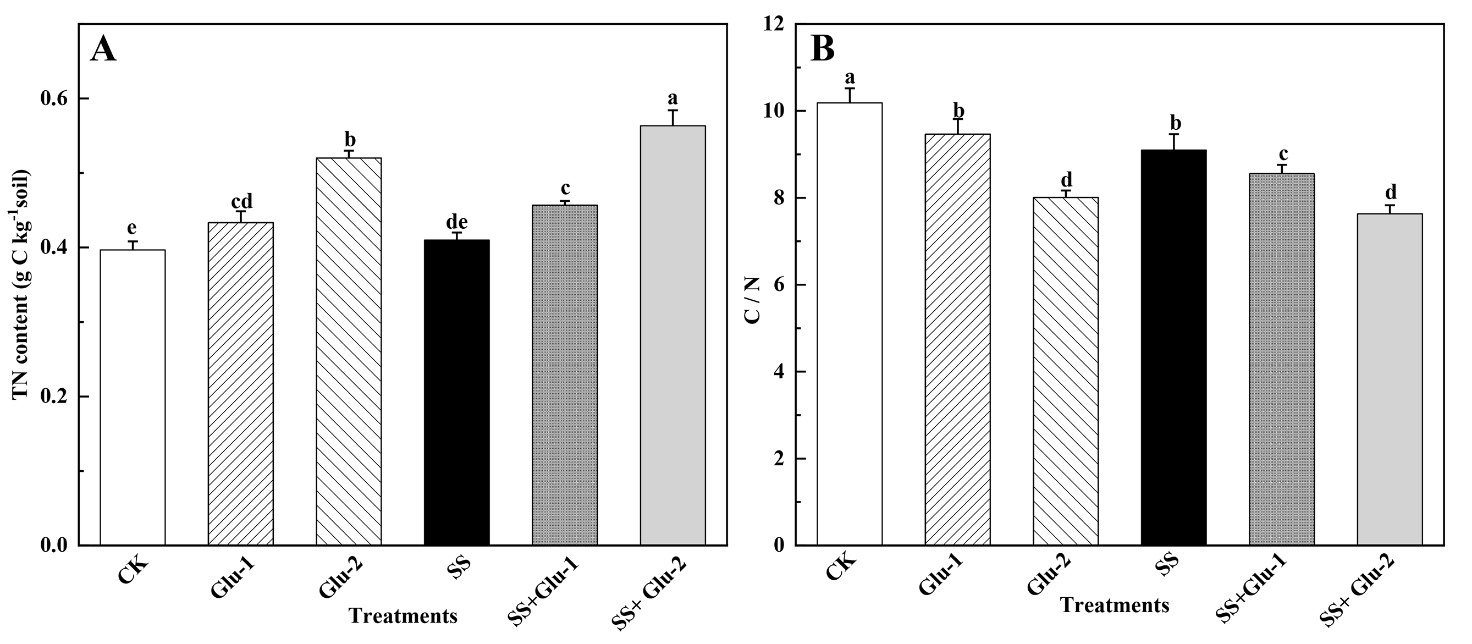


**S2 Fig. Content of total nitrogen (TN) of soil (A) and ratio of total soil organic carbon to TN (C/N, B) in the sterilized and non-sterilized soils with glucose addition at day 45.** CK, non-sterilized soil without glucose addition; Glu-1, non-sterilized soil with low level of glucose addition; Glu-2, non-sterilized soil with high level of glucose addition; SS, sterilized soil without glucose addition; SS+Glu-1, sterilized soil with low level of glucose addition; SS+Glu-2, sterilized soil with high level of glucose addition. Different lowercase letters indicate significant differences (*P* < 0.05) among treatments.
