# Supplementary material for: Glucose addition promotes C fixation and bacteria diversity in C-poor soils, improves root morphology, and enhances key N metabolism in apple roots
Source: PLoS One. 2022 Jan 19;17(1):e0262691. doi: 10.1371/journal.pone.0262691 (PMC8773054; doi:10.1371/journal.pone.0262691)
Supplement: S3 Fig — δ13C values of soil organic carbon (A), microbial biomass carbon (B), water soluble organic carbon (C) and particulate organic carbon (D) in the sterilized and non-sterilized soils with glucose addition. Different uppercase letters indicate significant differences (P < 0.05) among different treatments at the same sampling time. Different lowercase letters indicate significant differences (P < 0.05) among different sampling time within the same treatment. Overlapping date points with the same significant differences are indicated by common letters. CK, non-sterilized soil without glucose addition; Glu-1, non-sterilized soil with low level of glucose addition; Glu-2, non-sterilized soil with high level of glucose addition; SS, sterilized soil without glucose addition; SS+Glu-1, sterilized soil with low level of glucose addition; SS+Glu-2, sterilized soil with high level of glucose addition. (DOCX) [file pone.0262691.s003.docx]

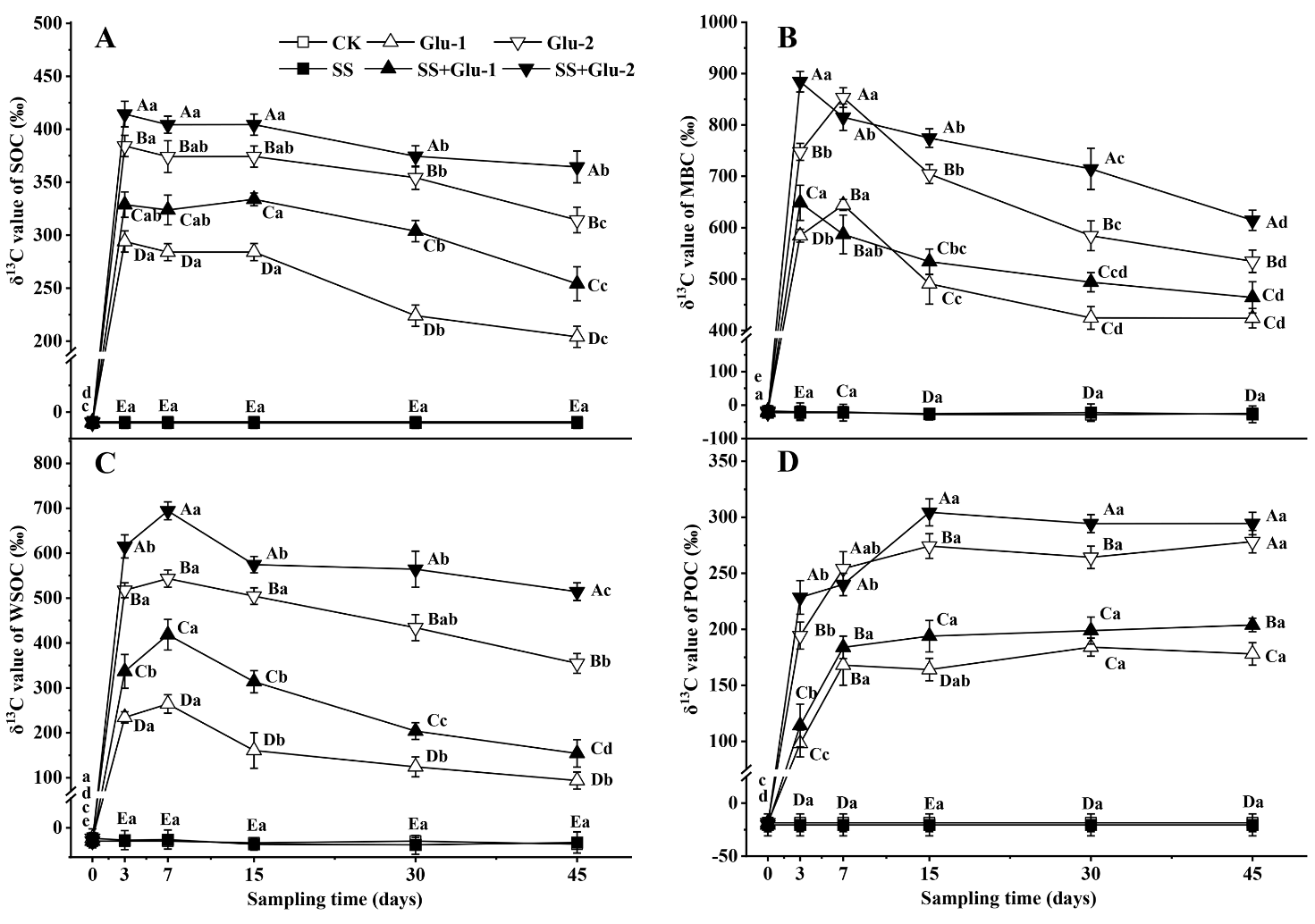


**S3 Fig. δ^13^C values of soil organic carbon (A)**, **microbial biomass carbon (B)**, **water soluble organic carbon (C) and particulate organic carbon (D) in the sterilized and non-sterilized soils with glucose addition**. Different uppercase letters indicate significant differences (*P* < 0.05) among different treatments at the same sampling time. Different lowercase letters indicate significant differences (*P* < 0.05) among different sampling time within the same treatment. Overlapping date points with the same significant differences are indicated by common letters. CK, non-sterilized soil without glucose addition; Glu-1, non-sterilized soil with low level of glucose addition; Glu-2, non-sterilized soil with high level of glucose addition; SS, sterilized soil without glucose addition; SS+Glu-1, sterilized soil with low level of glucose addition; SS+Glu-2, sterilized soil with high level of glucose addition.
