# Supplementary material for: Glucose addition promotes C fixation and bacteria diversity in C-poor soils, improves root morphology, and enhances key N metabolism in apple roots
Source: PLoS One. 2022 Jan 19;17(1):e0262691. doi: 10.1371/journal.pone.0262691 (PMC8773054; doi:10.1371/journal.pone.0262691)
Supplement: S5 Fig — Contents of NO3—N (A), NO2—N (B) and NH4+-N (C) of apple root in the sterilized and non-sterilized soils with glucose addition at day 45. Different lowercase letters indicate significant differences between treatments (P < 0.05). CK, non-sterilized soil without glucose addition; Glu-1, non-sterilized soil with low level of glucose addition; Glu-2, non-sterilized soil with high level of glucose addition; SS, sterilized soil without glucose addition; SS+Glu-1, sterilized soil with low level of glucose addition; SS+Glu-2, sterilized soil with high level of glucose addition. (DOCX) [file pone.0262691.s005.docx]

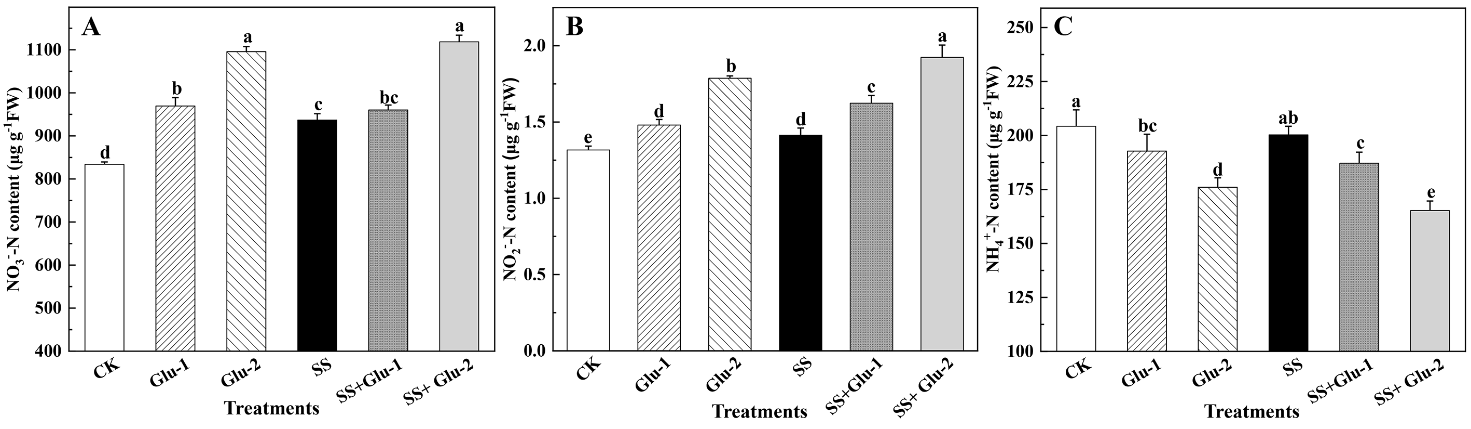


**S5 Fig. Contents of NO_3_^-^-N (**A**), NO_2_^-^-N (**B**) and NH_4_^+^-N (**C**) of apple root in the sterilized and non-sterilized soils with glucose addition at day 45**. Different lowercase letters indicate significant differences between treatments (*P* < 0.05). CK, non-sterilized soil without glucose addition; Glu-1, non-sterilized soil with low level of glucose addition; Glu-2, non-sterilized soil with high level of glucose addition; SS, sterilized soil without glucose addition; SS+Glu-1, sterilized soil with low level of glucose addition; SS+Glu-2, sterilized soil with high level of glucose addition.
