# Supplementary material for: Glucose addition promotes C fixation and bacteria diversity in C-poor soils, improves root morphology, and enhances key N metabolism in apple roots
Source: PLoS One. 2022 Jan 19;17(1):e0262691. doi: 10.1371/journal.pone.0262691 (PMC8773054; doi:10.1371/journal.pone.0262691)
Supplement: S1 Table — CK, non-sterilized soil without glucose addition; Glu-1, non-sterilized soil with low level of glucose addition; Glu-2, non-sterilized soil with high level of glucose addition; SS, sterilized soil without glucose addition; SS+Glu-1, sterilized soil with low level of glucose addition; SS+Glu-2, sterilized soil with high level of glucose addition. (DOCX) [file pone.0262691.s008.docx]

**S1 Table.** **Gene-specific primers** **used for quantitative real-time PCR in the sterilized and non-sterilized soils with glucose addition.**

| Gene Name | Primer-Reverse | Primer-Forward |
| --- | --- | --- |
| *NR* | CGCTTTGCGAAGTGCTGA | CCGATGAACCCCGGTATTA |
| *GS* | GGCTGTTGGCGATGTTAC | CACCCTTGGCTTGTTCTATT |
| *NADH-GDH* | ACCAACTTACGGGACAGC | GAACGCCATACGACAAGAG |
| *NADH-GOGAT* | GCTGGAGGCAACTAGACCTG | TTAGCACTCAGACGCCATTG |

CK, non-sterilized soil without glucose addition; Glu-1, non-sterilized soil with low level of glucose addition; Glu-2, non-sterilized soil with high level of glucose addition; SS, sterilized soil without glucose addition; SS+Glu-1, sterilized soil with low level of glucose addition; SS+Glu-2, sterilized soil with high level of glucose addition.
