# Supplementary material for: Glucose addition promotes C fixation and bacteria diversity in C-poor soils, improves root morphology, and enhances key N metabolism in apple roots
Source: PLoS One. 2022 Jan 19;17(1):e0262691. doi: 10.1371/journal.pone.0262691 (PMC8773054; doi:10.1371/journal.pone.0262691)
Supplement: S2 Table — Different lowercase letters indicate significant differences between treatments (P < 0.05). CK, non-sterilized soil without glucose addition; Glu-1, non-sterilized soil with low level of glucose addition; Glu-2, non-sterilized soil with high level of glucose addition; SS, sterilized soil without glucose addition; SS+Glu-1, sterilized soil with low level of glucose addition; SS+Glu-2, sterilized soil with high level of glucose addition. (DOCX) [file pone.0262691.s009.docx]

**S2 Table. Change in amino acid contents of root in the sterilized and non-sterilized soils with glucose addition at day 45.**

| Amino acid (μg g^-1^) | CK | Glu-1 | Glu-2 | SS | SS+Glu-1 | SS+Glu-2 |
| --- | --- | --- | --- | --- | --- | --- |
| Aspartic | 2425.8±7.9 e | 2439. 7±16.47 de | 2549.7±16.4 b | 2464.7±12.6 d | 2512.6±13.7 c | 2649.3±19.2 a |
| Glutamic | 2188.1±8.2 d | 2308.9±22.70 c | 2464.7±16.7 a | 2289.5±18.2 c | 2402.9±19.3 b | 2491.3±14.9 a |
| Threonine | 1325.4±9.1 c | 1345. 9±5.99 b | 1376.7±5.1 ab | 1352.5±10.7 b | 1346.5±14.3 b | 1382.9±8.1 a |
| Lysine | 1082.6±12.5 c | 1093.3±12.08 bc | 1171.9±12.1 ab | 1099.9±12.7 bc | 1124.6±9.0 b | 1180.6±20.0 a |
| Glycine | 1022.6±12.9 b | 1028.7±9.25 b | 1101.5±20.5 ab | 1038.9±19.1 b | 1056.9±25.4 b | 1129.9±15.0 a |
| Serine | 960.0±5.7 c | 983.6±11.87 bc | 1020.9±14.7 ab | 978.4±14.5 c | 1017.1±26.3 ab | 1040.8±24.2 a |
| Arginine | 857.3±8.6 c | 873.3±13.48 c | 939.9±11.9 a | 871.5±11.5 c | 903.4±13.2 b | 947.3±10.1 a |
| Histidine | 845.2±10.3 d | 850.5±2.98 cd | 884.4±10.5 a | 864.6±7.8 bc | 882.0±17.5 ab | 894.5±10.3 a |
| Alanine | 744.8±4.92 d | 774.1±9.21 c | 816.7±7.1 a | 771.2±6.9 c | 792.1±10.7 b | 823.2±7.5 a |
| Leucine | 591.8±14.1 d | 597.4±10.73 cd | 642.5±13.9 a | 614.1±8.8 bc | 621.9±9.3 b | 645.7±10.1 a |
| Proline | 487.2±7.2 c | 500.4±2.35 c | 516.3±9.5 ab | 498.9±8.5 c | 502.2±15.0 bc | 519.7±6.3 a |
| Phenylalanine | 403.6±11.6 c | 431.1±8.86 b | 465.0±7.1 ab | 439.7±9.7 b | 445.2±9.3 b | 478.6±5.2 a |
| Methionine | 298.1±5.1 d | 307.3±2.07 cd | 338.1±9.6 a | 311.3±3.1 bc | 319.9±1.8 b | 339.8±9.4 a |
| Valine | 206.4±6.5 c | 213.4±7.18 c | 236.2±3.2 ab | 218.2±7.7 b | 226.9±7.2 ab | 241.1±7.4 a |
| Tyrosine | 126.3±5.4 d | 148.1±3.20 c | 173.4±9.2 b | 145.8±7.1 c | 167.1±9.6 b | 189.2±10.1 a |
| Cysteine | 114.8±10.9 d | 117.9±4.59 d | 148.8±6.5 ab | 126.9±3.9 cd | 135.7±6.9 bc | 161.5±9.4 a |

Different lowercase letters indicate significant differences between treatments (*P* < 0.05). CK, non-sterilized soil without glucose addition; Glu-1, non-sterilized soil with low level of glucose addition; Glu-2, non-sterilized soil with high level of glucose addition; SS, sterilized soil without glucose addition; SS+Glu-1, sterilized soil with low level of glucose addition; SS+Glu-2, sterilized soil with high level of glucose addition.
