# Supplementary material for: Glucose addition promotes C fixation and bacteria diversity in C-poor soils, improves root morphology, and enhances key N metabolism in apple roots
Source: PLoS One. 2022 Jan 19;17(1):e0262691. doi: 10.1371/journal.pone.0262691 (PMC8773054; doi:10.1371/journal.pone.0262691)
Supplement: S3 Table — Different lowercase letters indicate significant differences between treatments (P < 0.05). CK, non-sterilized soil without glucose addition; Glu-1, non-sterilized soil with low level of glucose addition; Glu-2, non-sterilized soil with high level of glucose addition; SS, sterilized soil without glucose addition; SS+Glu-1, sterilized soil with low level of glucose addition; SS+Glu-2, sterilized soil with high level of glucose addition. (DOCX) [file pone.0262691.s010.docx]

**S3 Table. Change in organic acid contents of root in the sterilized and non-sterilized soils with glucose addition at day 45.**

| Organic acid  (μg g^-1^FW) | CK | Glu-1 | Glu-2 | SS | SS+Glu-1 | SS+Glu-2 |
| --- | --- | --- | --- | --- | --- | --- |
| α-Ketoglutaric acid | 2.13±0.1 c | 2.49±0.2 b | 3.14±0.24 a | 2.41±0.23 bc | 2.74±0.04 b | 3.22±0.2 a |
| Fumaric acid | 13.7±0.6 d | 15.2±0.4 bc | 16.6±0.35 a | 14.6±0.68 c | 15.1±0.40 b | 17.4±0.3 a |
| Succinic acid | 0.38±0.02 e | 0.41±0.03 de | 0.45±0.03 b | 0.51±0.03 d | 0.74±0.02 c | 0.74±0.02 a |
| Citric acid | 268.9±2.6 d | 274.2±5.6 d | 315.6±3.3 a | 282.6±4.6 c | 288.8±5.5 c | 305.7±4.9 b |
| Malic acid | 259.7±5.9 c | 270.1±4.8 b | 288.1±5.5 a | 268.8±6.7 bc | 275.1±5.7 b | 288.1±4.3 a |

Different lowercase letters indicate significant differences between treatments (*P* < 0.05). CK, non-sterilized soil without glucose addition; Glu-1, non-sterilized soil with low level of glucose addition; Glu-2, non-sterilized soil with high level of glucose addition; SS, sterilized soil without glucose addition; SS+Glu-1, sterilized soil with low level of glucose addition; SS+Glu-2, sterilized soil with high level of glucose addition.
