# Supplementary material for: Glucose addition promotes C fixation and bacteria diversity in C-poor soils, improves root morphology, and enhances key N metabolism in apple roots
Source: PLoS One. 2022 Jan 19;17(1):e0262691. doi: 10.1371/journal.pone.0262691 (PMC8773054; doi:10.1371/journal.pone.0262691)
Supplement: Attachment — Submitted filename: Response to Reviewers.docx [file pone.0262691.s011.docx]

**Responses to Comments**

**Reviewer #1:**

The manuscript presents the potential to be published in PLOS ONE Journal. Please consider the questions raised below.

Abstract p.2 l.23: Present the meaning of the acronym SOC the first time it appears in the text.

**Answer: We changed “SOC” to “soil organic C (SOC)” in Line 23 because the acronym appeared the first time in the manuscript.**

Abbreviations p.3 l.42: Insert meaning of acronyms when they first appear in the text.

**Answer: We substituted “****Low C soil” to “Low carbon soil”, “Exogenous C” to “Exogenous carbon”, and “N metabolism” to “Nitrogen metabolism” in Line 41.**

Materials and Methods p.9 l.193: Explain better the steps of DNA extraction, sequencing, and data analysis (How did you check the quality of the extracted DNA? Where and how was the Illumina sequencing performed? What are the steps of data analysis using bioinformatics?).

**Answer: The detailed steps of DNA extraction, sequencing, and data analysis were shown as follows:**

**Genomic DNA was extracted from soil samples at 45-day using a FastDNA Spin Kit (Omega Bio-Tek, Norcross, GA, USA) according to the manufacturer’s protocol. The quality of DNA was analysed with 1% agarose gel electrophoresis and the total quantity of DNA was determined using a Thermo NanoDrop 2000 UV Microvolume Spectrophotometer (Thermo Fisher Scientific, USA).** **The primers 338F (5'-ACTCCTACGGGAGGCAGCAG-3') and 806R (5'-GGACTACHVGGGTWTCTAAT-3') were chosen to amplify the 16S rRNA genes in the V3-V4 regions [49]. The PCR amplification conditions included an initial denaturation at 95 ℃ for 3 min, followed by 27 cycles of denaturation at 95 ℃ for 30 s, annealing at 60 ℃ for 30 s, extension at 72 °C for 30 s, and a finial extension at 72 ℃ for 10 min.** **The PCR products of all samples were purified with a Cycle Pure Kit (OMEGA), pooled in equimolar concentrations and performed on an Illumina (2 × 300 bp) MiSeq machine (Illumina, San Diego, CA, USA)** **at the Shanghai Origingene Biotechnology Co. Ltd., China.**

**The paired-end reads were analysed statistically by Trimmomatic software after depletion of primers. Bases of reads with a tail mass of 20 bp or less, overlapping paired-end reads less than 10 bp, and box sequences at both ends of reads were filtered. The unmatched sequences and singletons were** **excluded according to the Silva reference database v128 [50]. The operational taxonomic units were defined by clustering nonrepetitive sequences at 97% similarity and classified according to the Silva reference database using the Ribosomal Database Project Bayesian algorithm classifier (RDP) [51]. Then, Usearch version 7.1 was used to cluster the sequences with 97% similarity for operational taxonomic units (OTU) [52].**

**Difference in the composition of bacterial OTUs according to taxonomic category between treatments was assessed.** **After** **centred-log ratio (*clr*) transformation (log transformation of the geometric mean),** **the ‘*codaSeq.clr*’ function was used in the ‘*CoDaSeq*’ package of R software [53].** **The alpha diversity indices of bacterial communities, including ACE, Shannon and Simpson, were analysed using ‘*phyloseq*’ package of R software. Principal coordinate analysis (PCoA) and redundancy analysis (RDA) were performed using ‘*stats*’ and ‘*vegan*’ packages in R software [54], respectively.**

**We also added the detailed steps of DNA extraction, sequencing, and data analysis in Line 203-229 of the Materials and Methods Section.** **We also added the new references in the “References” section. The cited references in Materials and Methods were shown as follows:**

**49. Fadrosh DW, Ma B, Gajer P, Sengamalay N, Ott S, Ravel J, et al. An improved dual-indexing approach for multiplexed 16s rRNA gene sequencing on the Illumina Miseq platform. Microbiome. 2014;2:1-7. doi: 10.1186/2049-2618-2-6**

**50. Quast C, Pruesse E, Yilmaz P, Gerken J, Schweer T, Yarza P, et al. The SILVA ribosomal RNA gene database project: improved data processing and web-based tools. Nucleic Acids Res. 2012;41(D1):D590-D596. doi: 10.1093/nar/gks1219**

**51. Wang Q, Garrity GM, Tiedje JM, Cole JR. Naive Bayesian classifier for rapid assignment of rRNA sequences into the new bacterial taxonomy. Appl. Environ. Microbiol. 2007;73(16):5261-5267. doi: org/10.1128/AEM.00062-07**

**52. Edgar RC. Search and clustering orders of magnitude faster than BLAST. Bioinformatics, 2010;26(19):2460-2461. doi.org/10.1093/bioinformatics/btq461**

**53. Gloor GB and Reid G. Compositional analysis: a valid approach to analyze microbiome high-throughput sequencing data. Can. J. Microbiol. 2016;62(8):692-703. doi.org/10.1139/cjm-2015-0821**

**54. Dixon P. VEGAN, a package of R functions for community ecology. J. Veg. Sci. 2003, 14(6): 927-930. doi.org/10.1111/j.1654-1103.2003.tb02228.x**

Materials and Methods p.9 l.193: You must submit the raw FASTQ files from the sequencing to NCBI and inform the study accession number.

**Answer: The raw FASTQ files were deposited in the National Center for Biotechnology Information (NCBI)** **and the Sequence Read Archive (SRA) number was PRJNA765206. Please see Line 230.**

Materials and Methods p.9 l.197: Separate "Miseqmachine".

**Answer: We changed “Miseq****machine” to “Miseq machine” in Line 213.**

Materials and Methods p.10 l.202: Insert the term "analytical balance".

**Answer: We changed “The root was oven-dried for 24 h at 80 ℃ for dry weight analysis” into “****The root was oven-dried for 24 h at 80 ℃, and weighed with electronic analytical balance” in Line 236.**

Result p.12 l.251: Replace for "Results".

**Answer: We changed “Result” to “Results” in Line 278. Thanks for your careful review.**

Result p.14 l.319: I didn't find Good coverage results (Good, 1953). I suggest presenting these data, which demonstrate the quality of the sequencing.

**Answer: Thanks for your professional suggestion. We added the coverage data in Table 1 so as to demonstrate the quality of the sequencing. The coverage exceeded 0.994 in all treatments. Please see Line 342 and Table 1.**

Result p.14 l.319: Why has soil bacterial diversity not been analyzed? (beta and alpha diversity?). These analyzes contribute to the understanding of the structural dynamics of the community after the application of treatments.

**Answer: We analyzed soil bacterial alpha diversity indices (****including Chao, ACE, Shannon, Simpson) and beta diversity in order to understand the contribution of soil sterilization and glucose addition to the structural dynamics of the community. The values of Ace, Chao and Shannon indices were increased with the glucose addition levels, whereas the Simpson index showed the opposite trend. Compared with non-sterilized soil, sterilized soil with high level of glucose addition increased the values of Ace, Chao and Shannon indices by 13.8%, 7.9% and 11.4%, respectively; and sterilized soil with low level of glucose addition increased them by 17.3%, 13.8% and 2.6%, respectively; The** **PCoA plot based on the *clr*-transformed data was used to presented the changes of bacterial community structures in soil.** **The first two principal components explained 78.9% of total variations in the composition of bacterial communities (Fig 3A). The PCoA1 clearly separated the treatments with and without glucose addition. The non-sterilized and sterilized treatments were differentiated along the PCoA2. As presented by the** **hierarchical cluster analysis, bacterial communities revealed two clusters comprising samples from all treatment groups (Fig 3B). The treatments of non-sterilized soil and sterilized soil with the same level of glucose addition clustered together.**

**We added these data about alpha and beta diversity in Table 1 and Figure 3 and these results in Line 342-392.**

**We also added the discussion on the contribution of soil sterilization and glucose addition to soil bacterial communities in Line 526-561. Thanks for your professional comments.**

Result p.14 l.319: I raise a reflection on relative abundance results: There is an ongoing debate about the downside of interpreting sequencing data based on its relative abundance (which can cause data distortions). When we convert data into relative abundances, the independent variable appears to be correlated, since the frequency of any microbial community must add 1. To solve this problem, it is more feasible to use mathematical approaches for data composition analysis. The most accepted approach so far is Aitchison's centered logarithmic ratio transformation (*clr*).

**Answer: Thanks for your professional suggestion. We totally agreed with you. We** **log-transformed the data of soil bacterial communities.** **After centred-log ratio (*clr*) transformation (****log transformation of the geometric mean), the ‘*codaSeq.clr*’ function was used in the ‘*CoDaSeq*’ package of R software. Please see Line 223-229.**

Result p.14 l.319: A differential abundance analysis would also be interesting to highlight soil bacterial genera affected by the treatments.

**Answer: We added the difference analysis of relative abundance of bacterial community at the genera level among treatments. “****The** **heatmap of soil** **bacterial genera showed that all samples were clustered into two groups consisting of the treatment** **with glucose addition and that without glucose addition (Fig 3D). The relative abundances of members of *Proteobacteria* (*Pseudomonas*, *Skermanella* and *Acidibacter*,) *Firmicutes* (*Paenibacillus* and *Trichococcus*) and *Actinobacteria* (*Arthrobacter*, *Sinomonas* and *Blastococcus*) were higher, and those of *Proteobacteria* (*Mesorhizobium* and *Massilia*), *Chloroflexi* (*Caldilineaceae_norank*), *Acidobacteria* (*Bryobacter*) and *Actinobacteria* (*Acidothermus*) were lower in the SS+Glu-2 treatment relative to Glu-2 treatment. Moreover, the relative abundances of members of *Proteobacteria* (*Pseudomonas*) and *Firmicutes* (*Bacillus*) were higher, and those of *Actinobacteria* (*Blastococcus* and *Sinomonas*) and *Chloroflexi* (*Caldilineaceae_norank*) were lower in the SS treatment than those in the CK treatment.” Please see Line 383-392.**

Result p.14 l.319: Improve the description of phyla and genera found in each treatment (text is confusing).

**Answer: We rewrote the description about the difference analysis of relative abundances of bacterial communities at the phylum and genera levels among treatments. Please see Line 375-380 and Line 383-392. The detailed revision was shown as follows: The predominant bacterial phyla in all treatments were** ***Proteobacteria*, *Actinobacteria*, and *Acidobacteria*, with relative abundances larger than 10% (Fig 3C). The glucose addition enhanced the relative abundances of *Proteobacteria, Actinobacteria* and *Firmicutes* by 59.4%, 19.6% and 43.3%, but decreased those of *Acidobacteria* and *Chloroflexi* by 41.8% and 39.1%, respectively. The relative abundances of *Proteobacteria*, *Actinobacteria* and *Verrucomicrobia* were higher in the SS treatment than those in the CK treatment.**

Result p.14 l.339: In general, clearly demonstrate which treatment presented the best results (text is confusing).

**Answer: We rewrote the results about root morphology and biomass in order to make them clear. Please see Line 394-400. The detailed revision was showed as follows:**

**The root morphology indices (including surface area, volume, and length) were enhanced with the levels of glucose addition (Fig 4). Under the same level of glucose addition, root surface and volume were not significantly different (*****P*>0.05) between sterilized soil and non-sterilized soil (Figs 4A and 4B).** **The SS+Glu-2 treatment significantly increased (*P*<0.05) the total root length by 5.9% compared with Glu-2 treatment (Fig 4C). The sterilized and non-sterilized soils with glucose addition increased the root biomass, on average, by 23.2% and 25.4% compared with those without glucose addition, respectively (Fig 4D).**

Discussion p.22 l.430: Explore further the effect of soil sterilization on your bacterial community structure beyond the addition of glucose.

**Answer: We further discussed the effect of soil sterilization on bacterial community structure in Line 544-561 of the Discussion section. “The composition of bacterial communities at the phylum level was similar, while their relative abundances were different between sterilized and non-sterilized soils. Autoclave sterilization for short term (4 h) kills most of native soil microorganisms, leaving many empty niches for recolonized microbe to fill.** **On the other hand, plant growth could favor the** **recolonization of highly** **desirable microorganisms, which are used to assimilate root exudates as “food source”, once the niches competition was removed via sterilization [42, 91]. Therefore, microorganism occupied empty niches in the sterilized soils planted with apple seedlings. *Pseudomonas*, preferring root exudate C to the other C source, is beneficial bacteria for most plants [92]. This bacteria genus could firstly occupy empty niches in the sterilized soil, which may be the reason that *Pseudomonas* was more enriched in the SS treatment than those in the CK treatment. Regardless of sterilization or not, soil bacterial communities in the** **treatments without glucose addition were well separated from those in the treatments with glucose addition along the first component, which explained 60.05% of total variation (Fig 3A). This result indicated that the available C substrate supply plays dominate roles in bacterial communitie variation of low C soil. Moreover, the relative abundance of *Acidobacterium*, belonged to *Acidobacteria* (*K*-strategists), was higher than that *Bacillus*, belonged to *Firmicutes* (*r*-strategists), in the soil combined with sterilization and low level of glucose addition (Fig 3D). The *K*-strategists always dominates under low nutrients availability conditions, but are consistent with their outcompeting *r*-strategists when resources are limited [93, 94].”**

Discussion p.22 l.430: The contribution of soil bacteria to apple root growth remained to be discussed more. It would be interesting to correlate changes in community structure with candidate taxa for growth promotion.

**Answer: We further discussed the contribution of soil bacteria to apple root growth in Line 566-577. “The** **glucose addition and/or soil sterilization significantly increased the indices of root morphology. This was mainly associated with the increase in the relative abundance of *Burkholderia* (*Proteobacteria*), *Paenibacillus* (*Firmicutes*) and *Streptomyces* (*Actinobacteria*) by glucose addition and/or soil sterilization. The bacterial phyla of *Proteobacteria*, which consists mostly of G^-^ bacteria and diazotrophs,** **classified as plant growth promoting rhizobacteria (PGPR) [97, 98]. This PGPR exerts a beneficial effect on root growth on the production of phytohormone (e.g. IAA) or on nutrients uptake (e.g. N) by plants [99]. Some the other genera, such as *Streptomyces*, also have PGPR traits to hydrolyse chitin [100]. The genus *Paenibacillus* is easily isolated from the agricultural soil and rhizosphere [101] and has the characterise against pathogens [102]. Therefore, the above mentioned genera improved root morphology and promoted plant growth due to root hormones levels producing IAA in the rhizosphere and the nutrients availability.”**

Conclusion p.25 l.527: Better explain the applicability/projections of this study to field conditions.

**Answer: Thanks for your valuable suggestions. Now we have the highlight the intention and modified the conclusion (lines 616-628) in the following sentences:** **“****Glucose addition combined with soil sterilization not only increased the SOC content and new SOC formation derived from glucose C, but also increased the alpha and beta diversities of soil bacterial communities. Although soil microbial communities were similar between non-sterilization and sterilization, soil sterilization mainly increased the relative abundances of *Proteobacteria*, *Firmicutes* and *Verrucomicrobia* at the phyla level. Furthermore, the glucose addition, especially combined with soil sterilization improved root** **morphology, promoted the potential abilities of root N metabolism, and increased the amino acid synthesis in root. Overall, these results suggested the supply of C substrate with heathy soil conditions** **well shapes soil microbial communities and root morphology, and potentially increases soil C sequestration in agroecosystems. However, the complexity of C substrate drives the function and structure of soil microbial communities, which could lead to dynamics of plant growth and soil nutrient transformation.** **Further research should be focused on the coupled mechanism among nutrients transformation, plant growth and soil C sequestration under supply of complicated C substrates for low C soil.”**

Reviewer: 2

The paper entitled “Glucose addition promotes C fixation and bacteria community composition in C-poor soils, improves root morphology, and enhances key N metabolism in apple roots” reports an interesting approach where the authors added glucose to the sterilized and non-sterilized soil. Then, they studied the response of the plant, soil C fractionation, and their bacterial composition. To publish in Plos One journal is necessary improve the following:

The manuscript careful language revision and standardize the use of American or British English.

**Answer: We made careful revision of English language. Dr. Tingting An visited the University of Tennessee for more than one years as a scholar, and published more than 7 SCI papers as the first author or corresponding author. Dr. An made substantial revisions to the manuscript in order to make it more readable. We hope that the revised manuscript is readable and meets the standards for publication.**

The authors should extend the discussion, including the dynamic of r and k microorganisms when glucose is added.

**Answer: We further discussed the dynamics of r and k microorganisms after the addition of glucose to soil. The glucose addition enhanced the relative abundances of specific bacterial communities (including *Proteobacteria*, *Actinobacteria* and *Firmicutes* at the phylum level) in the low C soil, which is C-limited for microbial growth relative to N nutrient. Both *Proteobacteria* and *Actinobacteria* as copiotrophic bacteria (*r*-strategists) have strong abilities to utilize labile organic C source [84, 85]. While the relative abundance of *Proteobacteria* exceeded to that of *Actinobacteria* at day 45. *Actinobacteria* taxa own few amounts of high affinity transporters to transport specific substrate, which leads to their saturated proliferation under C-poor condition. Additionally, *Actinobacteria* may strongly compete soil nutrients with *Proteobacteria* [86]. A negative correlation between *Acidobacteria* and total SOC was found (Fig 8), which could be attributed that high level of glucose addition may produce disorder osmotic and aberrant growth of *Acidobacteria* cells as oligotrophic bacteria [87, 88]. Please see Line527-538.**

The results section is long. I considered that some results can be in the supplementary material.

**Answer: Thanks for your valuable comments. We listed some results as supplementary material. Please see the file of supplementary material.**

Discuss the effect of C addition on C:N ratio and which is their relationship with the results observed.

**Answer: We further discussed the the effect of C addition on C:N ratio and which is their relationship with the results observed. “The increase in soil TN content drives the shift of dominant microbial growth strategies from *K*-to *r*-strategists [89, 90]. Soils with higher level of glucose addition had higher TN content and lower C/N ratio (S2 Fig). And C/N was negatively associated with *Proteobacteria*, *Actinobacteria* and *Firmicutes* (opportunistic bacteria, *r*-strategists) (Fig 8). These results demonstrated that *r*-strategy decomposers rather than *K*-strategists dominate at lower substrate C/N ratios.” Please see Line538-543.**

The manuscript should show what is the importance of these results to agriculture.

**Answer: Now we have the highlight the intention and modified the conclusion (lines 616-628) in the following sentences:**

**“Glucose addition combined with soil sterilization not only increased the SOC content and new SOC formation derived from glucose C, but also increased the alpha and beta diversities of soil bacterial communities. Although soil microbial communities were similar between non-sterilization and sterilization, soil sterilization mainly increased the relative abundances of *Proteobacteria*, *Firmicutes* and *Verrucomicrobia* at the phyla level. Furthermore, the glucose addition, especially combined with soil sterilization improved root morphology, promoted the potential abilities of root N metabolism, and increased the amino acid synthesis in root. Overall, these results suggested the supply of C substrate with heathy soil conditions well shapes soil microbial communities and root morphology, and potentially increases soil C sequestration in agroecosystems. However, the complexity of C substrate drives the function and structure of soil microbial communities, which could lead to dynamics of plant growth and soil nutrient transformation. Further research should be focused on the coupled mechanism among nutrients transformation, plant growth and soil C sequestration under supply of complicated C substrates for low C soil.”**

Specific comments:

Title: The term bacterial community is don't used correctly. Did the Glucose addition promote the bacterial community?

**Answer: We totally agreed with you that “bacterial community” is not suitably used. We added the data and results about soil bacterial diversity (beta and alpha diversity) in Lines 342-355, Table 1 found that glucose addition markedly increased the bacterial community diversities. We changed the title into “****Glucose addition promotes C fixation and bacteria diversity in** **C-poor soils, improves root morphology, and enhances** **key N metabolism in apple roots”. Thanks for your valuable comments.**

Abstract: Please describe the abbreviation before the first citation. For example, “SOC”.

**Answer: We changed “SOC” into “soil organic C (SOC)”. Please see Line 24.**

Abstract: Line 30-31: The authors should check the use of the term "richness". Did they calculate the richness indexes?

**Answer: Thanks for your professional comment. We added the values of Chao, Ace, and Shannon indices to describe bacteria community richness and diversity in Line 342-348 and Table 1 of the Results section. We found that the glucose addition increased the richness and diversity indices of soil bacterial community compared with** **no-glucose addition. We changed “Bacterial community richness” to “Bacterial community richness and diversity” in Line 31.**

Keywords: Avoid the use of abbreviations, for example: C and N.

**Answer: We substituted the abbreviation of C and N with “carbon” and “nitrogen” in the Keywords, in Line 41.**

Introduction: Line 74-78: For me is not clear, how in soil with N depletion the addition of C can increase the N absorption by the plant?

**Answer: The “nitrogen mining and competition” theory (presented by Fontaine et al., 2011) interprets that easily available C addition stimulates soil microbial growth in the rhizosphere, leading to the mining of additional N from soil organic matter (SOM) by soil microorganisms, that is, the production of some extracellular enzymes and thus enhancement of subsequent SOM decomposition (Fontaine et al., 2003). Living microorganisms require soil nutrients for their growths. Hence, the competition between plants and microorganisms mainly for N and phosphorus (P) in nutrient-limited soils (Kirkby et al., 2011). Microorganisms, as high surface area to volume ratios, show substantially faster initial uptake of all N forms (Fischer et al., 2010). However, the short life cycle of rhizosphere microbes and unidirectional N flux from soil to roots facilitates the transform of N from microorganisms to roots (Rosswall T, 1982; Schmidt et al., 2007). On the other hand, the addition of easily available C is depleted within a few days via microbial utilization and decomposition. The imbalance between absence of new C input and continuous consumption of old C by soil microorganisms leads to the release of N from microbial necromass into the soil, which results in the availability of N for plants (Yakov and Xu, 2013).**

**The following references have been used to support above explanation:**

**Fontaine S, Mariotti A, Abbadie L. The priming effect of organic matter: a question of microbial competition? Soil Biology and Biochemistry, 2003, 35(6): 837-843. doi: org/10.1016/S0038-0717(03)00123-8**

**Kirkby CA, Kirkegaard JA, Richardson AE, et al. Stable soil organic matter: a comparison of C:N:P:S ratios in Australian and other world soils. Geoderma, 2011, 163(3-4): 197-208. doi: org/10.1016/j.geoderma.2011.04.010**

**Fischer H, Ingwersen J, Kuzyakov Y. Microbial uptake of low‐molecular‐weight organic substances out‐competes sorption in soil. European Journal of Soil Science, 2010, 61(4): 504-513. doi: org/10.1111/j.1365-2389.2010.01244.x**

**Rosswall T. Microbiological regulation of the biogeochemical nitrogen cycle. Plant and Soil, 1982, 67(1): 15-34. doi: 10.1007/978-94-009-7639-9_2**

**Schmidt SK, Costello EK, Nemergut DR, et al. Biogeochemical consequences of rapid microbial turnover and seasonal succession in soil. Ecology, 2007, 88(6): 1379-1385. doi: org/10.1890/06-0164**

**Schimel JP, Weintraub MN. The implications of exoenzyme activity on microbial carbon and nitrogen limitation in soil: a theoretical model. Soil Biology and Biochemistry, 2003, 35(4): 549-563. doi: org/10.1016/S0038-0717(03)00015-4**

**Kuzyakov Y, Xu X. Competition between roots and microorganisms for nitrogen: mechanisms and ecological relevance. New Phytologist, 2013, 198(3): 656-669. doi: 10.1111/nph.12235**

**Hence, we have added more elaboration on the relationship between exogenous C addition and N absorption by root in introduction.** **We rewrote this paragraph and focused on microbial strategies responded to C substrate and soil nutrients in order to make it clear. Please see Line 74-83.**

Introduction: Line 79-80: Here the authors should approach the dynamic of r and k microorganisms depending of the C source complexity added?

**Answer: We add some comments on the dynamics of *r* and *K* microorganisms affected by the exogenous C source complexity. Please see Line 74-80.**

**“****Moreover,** **relative abundances of microorganism with different growth strategies are varied with** **soil nutrient environments, which would** **shape different soil microbial** **community structure [18]. In general,** ***K*-strategists have lower growth rates and higher substrate affinities. Conversely, *r*-strategists have higher growth rates, lower substrate affinities and preferentially assimilate labile C [19]. The supply of labile C leads to the succession of microorganism from *r*- to *K*-strategists, and this process mainly depends on nitrogen (N) captured by soil microorganisms [20].”**

Introduction: Line 83: Replace the term “biological desert”.

**Answer: We changed “biological desert” to “****leaves numerous empty niches for microorganism re-colonization”** **in order to make the sentence clear. Please see Line 87.**

Materials and methods: Line 125: Please, clarify the term “debris”.

**Answer: We firstly picked out** **the visible plant roots, rock pieces and the other debris from soil samples before experiment. We changed this sentence into “We picked out the visible plant root, rock pieces and the other debris from soil samples, passed them through a 2 mm sieve, and then fully mixed for pot experiments.” in order to make this sentence clear. Please see Line 130-131.**

Materials and methods: Line 127-129: Describe the references used to perform the chemical characterization of the soil.

**Answer: Thanks for your careful reading. We add the references used to perform soil basic properties in Line 135-138.** **The measured methods of SOC, total N, δ^13^C value, and MBC were showed in the following section, and the contents of available N, available P, available K, and pH value were analysed** **with the methods by Le and Marschner [37], and particle size separation was carried out with the method by Jensen et al. [38].**

Materials and methods: Line 145-146. The authors should have irrigated the two soils (sterilized and unsterilized) with sterile water.

**Answer: We really irrigated unsterilized soils with tap water so as to stimulate plant growth condition in fields (Qin et al.2014; Li et al. 2019; Moreira et al. 2019). We would irrigate unsterilized soil with sterile water to avoid the effect of irrigated water on soil microbial community in the future research. We added the cited references in Line 155.**

**Qin SJ, Zhou WJ, D Lyu, Liu LZ. Effects of soil sterilization and biological agent inoculation on the root respiratory metabolism and plant growth of *Cerasus sachalinensis* Kom. Sci. Hortic. 2014;170(1):189-195. doi: 10.1016/j.scienta.2014.03.019**

**Li K, DiLegge M J, Minas I S, et al. Soil sterilization leads to re-colonization of a healthier rhizosphere microbiome. Rhizosphere. 2019;12:100176. doi: 10.1016/j.rhisph.2019;100176**

**Moreira H, Pereira S I A, Marques A P G C, et al. Effects of soil sterilization and metal spiking in plant growth promoting rhizobacteria selection for phytotechnology purposes. Geoderma. 2019;334:72-81. doi: 10.1016/j.geoderma.2018.07.025**

Materials and methods: Line 146: change “injetected” for “applied”

**Answer: We changed “injetected” to “applied” in Line 154.**

Materials and methods: Line 150-153: The authors should clearly describe the soil collection conditions. Was the soil influenced by the roots?

**Answer:** **After glucose addition for 3, 7, 15, 30, and 45 days, soil samples were randomly collected from five pots with the similar seedling growth (one pot as one replication) per treatment. The aboveground seedings were firstly cut at the root base, and then the roots and soil cores remained in the pots were destructively collected. The soil samples adhered to root were carefully separated with shaking method because the seeding roots occupied the whole pots. After being removed the visible roots, the collected soil sub-samples were mixed thoroughly, and then were divided into half for further analysis.**

**The soil was influenced by the roots because the seedling root occupied the whole pot (internal diameter 10 cm, height 12 cm). We added the detailed conditions for soil sample collection. Please see Line 157-162.**

Materials and methods: Line 155: Clarify the statistical designs. The authors use 30 seedling roots per replicate or treatment.

**Answer: Thanks for your careful revision. We collected 5 pots (1 seedling per pot) per treatment, and set up 6 treatments. Hence, we collected 30 seedling roots (5 seedlings/treatment** × **6 treatments) in total on day 45. Sorry for our miswriting.**

Materials and methods: Line 166: change “rpm” for “× g”

**Answer: We changed 4000 rpm to 3000 × g in Line 175.**

Materials and methods: Line 193: The sequencing data should be deposited in a database.

**Answer: The raw FASTQ files were deposited in the National Center for Biotechnology Information (NCBI) and Sequence Read Archive (SRA) number was PRJNA765206. We added this information** **in Line 230-231. Thanks for your professional comments.**

Materials and methods: Line 193: Change “microbial” for “bacterial.

**Answer: We changed “Soil microbial community” to “DNA extraction, PCR amplification and bioinformatic analysis of bacteria” to make the sentence clear. Please see Line 202.**

Materials and methods: Line 193: In this section the authors should give more details of methodology used to analyze the data (bioinformatic analyses). How was performed the DNA extraction? for sequencing, did you use kit V2 or V3?

**Answer: We added the more details on DNA extraction, sequencing, and data analysis in Line 203-229. “Genomic DNA was extracted from soil samples at 45-day using a FastDNA Spin Kit (Omega Bio-Tek, Norcross, GA, USA) according to the manufacturer’s protocol. The quality of DNA was analysed with 1% agarose gel electrophoresis and the total quantity of DNA was determined using a Thermo NanoDrop 2000 UV Microvolume Spectrophotometer (Thermo Fisher Scientific, USA). The primers 338F (5'-ACTCCTACGGGAGGCAGCAG-3') and 806R (5'-GGACTACHVGGGTWTCTAAT-3') were chosen to amplify the 16S rRNA genes in the V3-V4 regions [49]. The** **PCR amplification conditions included an initial denaturation at 95 ℃ for 3 min, followed by 27 cycles of denaturation at 95 ℃ for 30 s, annealing at 60 ℃ for 30 s, extension at 72 °C for 30 s, and a finial extension at 72 ℃ for 10 min. The PCR products of all samples were purified with a Cycle Pure Kit (OMEGA), pooled in equimolar concentrations and performed on an Illumina (2 × 300 bp) MiSeq machine (Illumina, San Diego, CA, USA) at the Shanghai Origingene Biotechnology Co. Ltd., China.**

**The paired-end reads were analysed statistically by Trimmomatic software after depletion of primers.** **Bases of reads with a tail mass of 20 bp or less, overlapping paired-end reads less than 10 bp, and box sequences at both ends of reads were filtered. The unmatched sequences and singletons were excluded according to the Silva reference database v128 [50]. The operational taxonomic units were defined by clustering nonrepetitive sequences at 97% similarity and classified according to the Silva reference database using the Ribosomal Database Project Bayesian algorithm classifier (RDP) [51]. Then, Usearch version 7.1 was used to cluster the sequences with 97% similarity for operational taxonomic units (OTU) [52].**

**Difference in the composition of bacterial OTUs according to taxonomic category between treatments was assessed. After centred-log ratio (*clr*) transformation (log transformation of the geometric mean), the ‘*codaSeq.clr*’ function was used in the ‘*CoDaSeq*’ package of R software [53]. The alpha diversity indices of bacterial communities, including ACE, Shannon and Simpson, were analysed using ‘*phyloseq*’ package of R software. Principal coordinate analysis (PCoA) and redundancy analysis (RDA) were performed using ‘*stats*’ and ‘*vegan*’ packages in R software [54], respectively.”**

Materials and methods: Line 196: The authors should include the reference of primers used.

**Answer:** **Thanks for your thoughtful comments. We added the reference of primers used in Line 209. The cited reference was as following:**

**Fadrosh DW, Ma B, Gajer P, Sengamalay N, Ott S, Ravel J, et al. An improved dual-indexing approach for multiplexed 16s rRNA gene sequencing on the Illumina Miseq platform. Microbiome. 2014; 2:1-7. doi: 10.1186/2049-2618-2-6**

Materials and methods: Line 204: The authors should include the reference of technique used.

**Answer: We added the reference of technique used in Line 234. The cited reference was as following:**

**Agapit C, Gigon A, Blouin M. Earthworm effect on root morphology in a split root system. Plant Biosyst. 2018; 152(4):780-786. doi: doi.org/10.1080/11263504.2017.1338627**

Materials and methods: Line 230-231: Please check the redaction.

**Answer: We revised the sentence into “****About 0.2 g frozen root sample and 2 mL reaction agent (****50 mmol L^-1^ Tris-HCl with pH 8.0, 2 mmol L^-1^ MgCl_2_, 2 mmol L^-1^ DTT and 0.4 mol L^-1^ sucrose) were fully homogenized” in order to make it clear. Please see Lines 252-253.**

Results: In the figures and tables presented, the authors should include the abbreviation definitions in legends.

**Answer:** **W****e added the abbreviation definitions in legends in all the figures and tables in order to make them clear. Please see all the figures and tables.**

Results: Line 255-258: Why does the glucose addition increases the SOC only in low doses?

**Answer: Exogenous C addition to sterilization soil can stimulate microorganisms increase, thereby promoting the increase of microbial biomass (Esch, et al. 2013). This increased microbial biomass can supply more microbial necromass for forming recalcitrant C and benefit SOC accumulation (Schmidt et al., 2011). To a certain extent, the quantity of activated microorganisms increased with the amount of labile C added. In our study, sterilization soil results in clay particles gathered and increase the surface and available sorption site to glucose-C. Hence, residual rate of glucose-C with higher level glucose in SS+Glu-2 treatment increase than Glu-2. Thereby actives more amounts of microorganisms and produces larger microbial biomass and prominently contribute to sequestration of SOC. But the lower level of glucose addition showed the opposite trend. Lower level of glucose-C addition resulted in actived microbe produces less microbial biomass and contribute to less sequestration of SOC in SS+Glu-1.**

**The following references have been used to support above explanation:**

**Esch, E. H., Hernandez, D. L., Pasari, J. R., Kantor, R. S. G., & Selmants, P. C. (2013). Response of soil microbial activity to grazing, nitrogen deposition, and exotic cover in a serpentine grassland. Plant and Soil, 366(1–2), 671–682. https://doi.org/10.1007/s11104-012-1463-5**

**Schmidt, M. W., Torn, M. S., Abiven, S., Dittmar, T., Guggenberger, G., Janssens, I. A., Trumbore, S. E. (2011). Persistence of soil organic matter as an ecosystem property. Nature, 478, 49–56. https://doi.org/10.1038/nature10386**

Results: Line 320: Please, analyze the data to family level.

**Answer:** **We presented the data on soil microbial community structure at the family level in S4 Fig and added the related results in Line 380-382.**

Results: Line 326-327: My suggestion is to compare with the data of soil after sterilization.

**Answer: We rewrote the results and focused on the comparison of data after sterilization. Please see Line 383-392. The detailed revisions were showed as follows:**

**The heatmap of soil bacterial genera showed that all samples were clustered into two groups consisting of the treatment with glucose addition and that without glucose addition (Fig 3D). The relative abundances of members of *Proteobacteria* (*Pseudomonas*, *Skermanella* and *Acidibacter*,) *Firmicutes* (*Paenibacillus* and *Trichococcus*) and *Actinobacteria* (*Arthrobacter*, *Sinomonas* and *Blastococcus*) were higher, and those of *Proteobacteria* (*Mesorhizobium* and *Massilia*), *Chloroflexi* (*Caldilineaceae_norank*), *Acidobacteria* (*Bryobacter*) and *Actinobacteria* (*Acidothermus*) were lower in the SS+Glu-2 treatment relative to Glu-2 treatment. Moreover, the relative abundances of members of *Proteobacteria* (*Pseudomonas*) and *Firmicutes* (*Bacillus*) were higher, and those of *Actinobacteria* (*Blastococcus* and *Sinomonas*) and *Chloroflexi* (*Caldilineaceae_norank*) were lower in the SS treatment than those in the CK treatment.**

Results: Line 340: Is curiously that root biomass increased in C.

**Answer: Thanks for your valuable comments. Root biomass increased in SS treatment may be attributed to the following reasons: (1) sterilization removes pathogen and alleviates disease suppression (Sosnowski et al., 2009; Savory, 1966). (2) the high temperature (121 ℃) and pressure (2 bar) during autoclaving affects the physical and chemical structure of the soil and liberates labile C, such as WSOC, and N (Mahmood et al., 2014; Berns et al., 2008), which enhances root development and acquisition of nutrients, then increases the root biomass (3) Since the original soils had been sterilized prior to reinoculation, the community assemblage recolonization phase was primarily influenced by the new habitat and the adequacy of its conditions for each of the inoculated taxon (Mallon et al., 2015), and increased availability of labile C and N (due to steam sterilization) may have influenced the new community formation by promoting the growth of copiotrophic microorganisms. Those copiotrophic microorganisms classified as plant growth promoting rhizobacteria (PGPR) (Dommelen et al, 2007; Ali et al, 2015), benefited root growth and nutrient absorption, thereby improved the biomass of root.**

**The following references have been cited to support above reasons:**

**Sosnowski MR, Fletcher JD, Daly AM, Rodoni BC, Viljanen-Rollinson SLH. Techniques for the**

**treatment, removal and disposal of host material during programmes for plant pathogen eradication. Plant Pathol. 2009;58(4):621-635. doi:10.1111/j.1365-3059.2009.02042.x**

**Savory BM. Specific replant diseases causing root necrosis and growth depression in perennial fruit and plantation crops. Farnham Royal. 1966 (1).**

**Marschner B, Bredow A. Temperature effects on release and ecologically relevant properties of dissolved organic carbon in sterilised and biologically active soil samples. Soil Biol. Biochem. 2002;**

**34:459e466. doi: 10.1016/S0038-0717(01)00203-6**

**Mahmood T, Mehnaz S, Fleischmann F, Ali R, Hashmi ZH, Iqbal Z. Soil sterilization effects on root growth and formation of rhizosheaths in wheat seedlings. Pedobiologia. 2014;57:123-130. doi: org/10.1016/j.pedobi.2013.12.005**

**Berns AE, Philipp H, Narres HD, Burauel P, Vereecken H, Tappe W. Effect of gamma-sterilization and autoclaving on soil organic matter structure as studied by solid state NMR, UV and fluorescence spectroscopy. Eur. J. Soil Sci. 2008;59:540-550. doi: 10.1111/j.1365-2389.2008.01016.x**

**Mallon CA, Poly F, LeRoux X, Marring I, vanElsas JD, Salles JF. Resource pulses can alleviate the biodiversity-invasion relationship in soil microbial communities. Ecology, 2015;96(4):915-926. doi: org/10.1890/14-1001.1**

**Dommelen AV, Vanderleyden J. Chapter 12: associative nitrogen fixation. In: The Biology of the Nitrogen Cycle. Elsiver B.V, 2007; pp.179-192. doi: 10.1016/B978-0-444-52857-5.X5000-0**

**Ali GS, Norman D, El-Sayed AS. Soluble and volatile metabolites of plant growth-promoting rhizobacteria (PGPRs). Adv. Bot. Res. 2015;75:241-284. doi: 10.1016/bs.abr.2015.07.004**

Results: Fig 10. What is the meaning the color in the correlation test.

**Answer:** **Thank you for your professional comments. Black arrow represents soil bacterial communities at the phylum level, red arrow represents soil nitrogen and organic carbon fractions, and green arrow represents root morphology indices. Circle and square denote the non-sterilized and sterilized soils, respectively.** **MBC, microbial biomass carbon; SOC, soil organic carbon;** **TN, total nitrogen; C/N, ratio of total soil organic carbon to TN; POC, particulate organic carbon; WSOC, water-soluble organic carbon; NH_4_^+^-N, ammonium nitrogen; NO_3_^-^-N, nitrate nitrogen; NO_2_^-^-N, nitrite nitrogen; RV, root volume; RS, root surface; RL, root length; RB, root biomass. Please see Fig 8.**

Discussion: Line 442-444: This paragraph is not clear, please rewriting.

**Answer: We rewrote this paragraph in order to make it clear. Please see Line 506-516. The detailed revision was showed as follows:**

**“The net SOC balance depends on the difference between new C gain and native SOC loss [73]. Glucose addition at low level leads to native SOC loss approximately equal to SOC gain in non-sterilized soil (Fig 2). Thus, the balance between accumulation and loss of SOC is probably associated with priming effect [21]. Glucose input to soil could activate dormant microorganisms, causing SOM decomposition [74].** **But,** **new SOC formation derived from glucose C could offset native SOC loss in the sterilized soil with low level of glucose addition. Moreover, the residual rate of glucose-C in the sterilized soil was higher than that in non-sterilized soil under the same level of glucose addition (Fig 1). These results demonstrated that soil sterilization may promote glucose-C** **sequestration in a low C soil. The process of autoclaving could destroy soil structure and lead to soil particulate finer [75, 76], which enhances available surface area and provide many sorption sites for glucose-C retention in soil [77].”**

Discussion: Line 444: The authors should not affirm that the glucose increased the “net C sequestration”.

**Answer: Thanks for your professional comments. The term of “net C sequestration” is not seriously used in this sentence. We changed the “net C sequestration” into “net SOC balance” in Line 506. In this research, net SOC sequestration equaled to the difference between native SOC and new SOC derived from glucose-C.**

Discussion: Line 477-479: How the addition and sterilization increase the indices of root morphology. I consider this result contradictory.

**Answer:** **Thanks for your suggestions. We really found that the glucose addition and sterilization increased the indices of root morphology and enhanced root biomass. The possible reasons are:** **The glucose addition and/or soil sterilization significantly increased the indices of root morphology. This was mainly associated with the increase in the relative abundance of *Burkholderia* (*Proteobacteria*), *Paenibacillus* (*Firmicutes*) and *Streptomyces* (*Actinobacteria*) by glucose addition and/or soil sterilization. The bacterial phyla of *Proteobacteria*, which consists mostly of G^-^ bacteria and diazotrophs, classified as plant growth promoting rhizobacteria (PGPR) [97, 98]. This PGPR exerts a beneficial effect on root growth on the production of phytohormone (e.g. IAA) or on nutrients uptake (e.g. N) by plants [99]. Some the other genera, such as *Streptomyces*, also have PGPR traits to hydrolyse chitin [100]. The genus *Paenibacillus* is easily isolated from the agricultural soil and rhizosphere [101] and has the characterise against pathogens [102]. Therefore, the above mentioned genera improved root morphology and promoted plant growth due to root hormones levels producing IAA in the rhizosphere and the nutrients availability. Those suggested the positive synergistic effect on root morphology between glucose addition and soil sterilization. Please see Line 566-577.**

Discussion: Line 490-491: The authors should cite the similar studies mentioned.

**Answer: We checked the references and substituted the references with the similar studies mentioned. Please see Line 551. The following references were showed as follows:**

**[92] Berendsen R L, Pieterse C, Bakker P. The rhizosphere microbiome and plant health. Trends Plant Sci. 2012;17(8):478-486. doi: 10.1016/j.tplants.2012.04.001**

Conclusions: The data presented does not permit conclude on changes in bacterial richness when applied glucose or/and sterilize the soil.

**Answer: Thanks for your professional comments. We added the data about the diversity and richness indices of soil bacterial community in Results section, Table 1 and Fig 3. “The values of Ace, Chao and Shannon indices were increased with the glucose addition levels, whereas the Simpson index showed the opposite trend. Compared with non-sterilized soil, sterilized soil with high level of glucose addition increased the values of Ace, Chao and Shannon indices by 13.8%, 7.9% and 11.4%, respectively; and sterilized soil with low level of glucose addition increased them by 17.3%, 13.8% and 2.6%, respectively.” and “****The PcoA plot based on the clr-transformed data was used to presented the changes of bacterial community structures in soil. As shown in Fig 3A, the total of 78.9% of variations in the composition of bacterial communities could be explained by the first two principal components. The PCoA1 clearly separate glucose addition samples from without addition samples. The CK and SS treatments were differentiated along the PcoA2, which demonstrated that soil sterilization was a main determinant on the second principal coordinate. For the same addition level, no-sterilized and sterilization soils formed a subgroup. As presented by the hierarchical cluster analysis, bacterial communities revealed two clusters comprising samples from all treatment groups (****Fig 3B). the samples of CK and SS were closer to each other and the samples of Glu-1, SS+Glu-1, Glu-2 and SS+Glu-2 were closer to each other.”**

**So, we rewrote the conclusion that “Glucose addition combined with soil sterilization not only increased the SOC content and new SOC formation derived from glucose C, but also increased the alpha and beta diversities of soil bacterial communities. Although soil microbial communities were similar between non-sterilization and sterilization, soil sterilization mainly increased the relative abundances of *Proteobacteria*, *Firmicutes* and *Verrucomicrobia* at the phyla level. Furthermore, the glucose addition, especially combined with soil sterilization improved root morphology, promoted the potential abilities of root N metabolism, and increased the amino acid synthesis in root.** **Overall, these results suggested the supply of C substrate with heathy soil conditions well shapes soil microbial communities and root morphology, and potentially increases soil C sequestration in agroecosystems. However, the complexity of C substrate drives the function and structure of soil microbial communities, which could lead to dynamics of plant growth and soil nutrient transformation. Further research should be focused on the coupled mechanism among nutrients transformation, plant growth and soil C sequestration under supply of complicated C substrates for low C soil.”. Please see Line 616-628.**

**Other revisions:**

**We updated references according to the added statement in the whole manuscript.**

**We hope these changes address all the concerns that you have with this manuscript. We look forward to your reply.**

**Sincerely,**

**Bianbin Qi and Deguo Lv on behalf of Sijun Qin**
