# Supplementary material for: Glucose addition promotes C fixation and bacteria diversity in C-poor soils, improves root morphology, and enhances key N metabolism in apple roots
Source: PLoS One. 2022 Jan 19;17(1):e0262691. doi: 10.1371/journal.pone.0262691 (PMC8773054; doi:10.1371/journal.pone.0262691)
Supplement: Attachment — Submitted filename: Response to Reviewers.docx [file pone.0262691.s012.docx]

**Dear Editor and Reviewers,**

**Ref.: PONE-D-21-22234R1**

**Glucose addition promotes C fixation and bacteria diversity in C-poor soils, improves root morphology, and enhances key N metabolism in apple roots**

**Responses to Comments**

**Reviewer #2:**

The authors should refer to the increase of α-diversity and changes in bacterial community structure (β-diversity. You should avoid the use of the phrase "increase of β-diversity". Please, check throughout the manuscript).

**Answer: We checked throughout the manuscript and changed “increased the alpha and beta diversities of soil bacterial communities” to “increased the alpha diversity and** **changed bacterial community structure in soils” in Line 618-619. Thanks for your suggestion.**

The authors sequenced V3-V4 16S rRNA region. They should refer to the bacterial community. Please, avoid the use of the microbial community throughout the manuscript.

**Answer: We checked the whole manuscript and changed “microbial community” to “****bacterial community” in Line 76, 83, 88 and 565. Thanks for your professional comments.**

The authors should change “heathy soil” for “healthy soil”. Please, check throughout the manuscript.

**Answer: Thanks for your careful revision. We have changed “heathy soil” to “healthy soil” in Line 624. Sorry for our miswriting.**

**Responses to Journal Requirements**

Please review your reference list to ensure that it is complete and correct. If you have cited papers that have been retracted, please include the rationale for doing so in the manuscript text, or remove these references and replace them with relevant current references. Any changes to the reference list should be mentioned in the rebuttal letter that accompanies your revised manuscript. If you need to cite a retracted article, indicate the article’s retracted status in the References list and also include a citation and full reference for the retraction notice.

**Answer: We thoroughly checked the reference list of the manuscript to ensure that it is complete and correct. All the cited references in paper have not been retracted. The reference NO. 65 in the previous version “**You YL, Wang JB, Shi WS, Pan DM. Extraction of genomic DNA of Litchi chinensis and optimizationof the RAPD reaction system. Biotechnology Bulletin. 2010;4113-115**” is published in a Chinses journal. We changed this reference to** “Gasic K, Hernandez A, Korban SS. RNA extraction from different apple tissues rich in polyphenols and polysaccharides for cDNA library construction” published in Plant Mol Biol Rep. 2004;22(4):437-438 (a SCI journal) **in order to make the citation rational. Please see Line 263.**

**We hope these changes address all the concerns that you have with this manuscript. We look forward to your reply.**

**Sincerely,**

**Bianbin Qi and Deguo Lv on behalf of Sijun Qin**
